# Nanocellulose/Nanoporous Silicon Composite Films as a Drug Delivery System

**DOI:** 10.3390/polym16142055

**Published:** 2024-07-18

**Authors:** Karla A. Garrido-Miranda, Héctor Pesenti, Angel Contreras, Judith Vergara-Figueroa, Gonzalo Recio-Sánchez, Dalton Chumpitaz, Silvia Ponce, Jacobo Hernandez-Montelongo

**Affiliations:** 1 Scientific and Technological Bioresource Nucleus (BIOREN-UFRO), Universidad de La Frontera, Temuco 4780000, Chile; 2 Núcleo de Investigación en Bioproductos y Materiales Avanzados (BioMA), Universidad Católica de Temuco, Temuco 4813302, Chile; hpesenti@uct.cl; 3 Departamento de Ciencias Biológicas y Químicas, Universidad Católica de Temuco, Temuco 4813302, Chile; acontrer@uct.cl; 4 Departamento de Ingeniería en Madera, Centro Biomateriales y Nanotecnología (CBN), Facultad de Ingeniería, Universidad del Bío-Bío, Concepción 4030000, Chile; jvergara@ubiobio.cl; 5 Departamento de Ingeniería Mecánica, Facultad de Ingeniería, Universidad del Bío-Bío, Concepción 4030000, Chile; 6 Grupo de Investigación en Materiales Avanzados (GIMAF), Universidad del Bío-Bío, Concepción 4030000, Chile; 7 Facultad de Ingeniería, Arquitectura y Diseño, Universidad de San Sebastián, Concepción 4080871, Chile; gonzalo.recio@uss.cl; 8 Facultad de Ciencias, Universidad Nacional de Ingeniería, Av. Túpac Amaru 210, Lima 15333, Peru; antonhyo2005@gmail.com; 9 Facultad de Ingeniería, Universidad de Lima, Av. Javier Prado Este 4600, Lima 15023, Peru; sponce@ulima.edu.pe; 10 Departamento de Bioingeniería Traslacional, Universidad de Guadalajara, Guadalajara 44430, Mexico

**Keywords:** nanocellulose, nanoporous silicon, composite material, drug delivery system

## Abstract

Nanocellulose (NC) is a promising material for drug delivery due to its high surface area-to-volume ratio, biocompatibility, biodegradability, and versatility in various formats (nanoparticles, hydrogels, microspheres, membranes, and films). In this study, nanocellulose films were derived from “Bolaina blanca” (*Guazuma crinita*) and combined with nanoporous silicon microparticles (nPSi) in concentrations ranging from 0.1% to 1.0% (*w*/*v*), using polyvinyl alcohol (PVA) as a binding agent to create NC/nPSi composite films for drug delivery systems. The physicochemical properties of the samples were characterized using UV-Vis spectroscopy, scanning electron microscopy (SEM), Fourier transform infrared spectroscopy–attenuated total reflectance (FTIR–ATR), X-ray diffraction (XRD), and thermogravimetric analysis (TGA). The mechanical properties and drug release capabilities were also evaluated using methylene blue (MB) as an antibacterial drug model. Antibacterial assays were conducted against *S. aureus* and *E. coli* bacteria. The results show that NC/nPSi composites with 1% nPSi increased the T_50%_ by 10 °C and enhanced mechanical properties, such as a 70% increase in the elastic modulus and a 372% increase in elongation, compared to NC films. Additionally, MB released from NC/nPSi composites effectively inhibited the growth of both bacteria. It was also observed that the diffusion coefficients were inversely proportional to the % nPSi. These findings suggest that this novel NC/nPSi-based material can serve as an effective controlled drug release system.

## 1. Introduction

Cellulose and its derivatives are among the most abundant, biodegradable, and renewable compounds on the planet [1]. The hydrophilic properties of cellulose and its high mechanical strength are due to the hydroxyl groups that are presented in its structure. These groups stabilize the cellulose chains through hydrogen bonds [1,2]. Cellulose has the unique property of permitting the isolation of materials with dimensions in the nanometer range (1–100 nm). This material is called nanocellulose (NC). These materials possess the excellent properties of cellulose as well as those associated with nanoscale materials, such as a large surface area. NC can be classified into three types based on the production methods used: bacterial nanocellulose (BNC), nanocrystalline cellulose (NCC), and nanofibrillated cellulose (NFC). NFC has an advantage over BNC and NCC as it can be produced on an industrial scale [3,4].

Due to its biocompatibility, bioavailability, mechanical strength, high surface-to-volume ratio, and low toxicity, NC has become a widely used material for drug delivery studies. The selection between BNC, NCC, and NFC will depend on the drug delivery system being designed. Kupnik et al. (2020) [5] published a review of NC in drug delivery, showing that it has been used for the delivery of ibuprofen, amoxicillin, methotrexate, ketorolac tromethamine, metronidazole, nadolol, and ketoprofen. NC is used in various formats, including micro and nanoparticles, hydrogels, microspheres, membranes, and films. These formats facilitate the administration of drugs both externally and internally. For instance, films and membranes are also applied for wound regeneration.

NC is a film-forming material that is often blended with other functional components, such as polymers or nanoparticles [6], to enhance its mechanical and sustained drug-release properties. Polyvinyl alcohol (PVA) is a polymer used in the biomedical area for its film-forming, non-toxic, biocompatible, biodegradable, highly crystalline, hydrophilic, and miscible properties. PVA contains hydroxyl groups that easily form hydrogen bonds within cellulose [7]. In addition, this polymer can be used for the delivery of hydrophilic and hydrophobic drugs, unlike nanocellulose which has difficulty adhering to hydrophobic drugs [8]. Colturato and Goveia (2022) [9] obtained a membrane using NC as a matrix and PVA as a binder. The membrane was loaded with vitamin D3, and the results showed that this biomaterial improved its mechanical properties. Additionally, the sustained release of vitamin D3 was observed for 30 min in an aqueous solution.

Nanoporous silicon (nPSi) is a biomaterial with various properties. Firstly, it has high porosity, which allows for a high degree of loading. Secondly, its pore size is adjustable between 5 and 150 nm, enabling it to load a wide range of molecules. Thirdly, it is biocompatible and degrades naturally in the human body into orthosilicic acid [Si(OH)_4_] (non-toxic). Finally, it has a high specific surface area (200–800 m^2^/g), which can be functionalized for controlled drug release [10,11,12,13]. Therefore, the combination of NC, nPSi (as microparticles), and PVA can provide new advantageous physical–chemical characteristics to obtain a new biomedical material in film format.

The aim of this study was to develop NC/nPSi composites using PVA as a binding agent in the format of films for the controlled release of drugs. NC derived from Bolaina blanca (*Guazuma crinita*) was used as the matrix. Bolaina blanca is a fast-growing Amazonian forest species with multiple uses [14]. NC/nPSi composites were produced through solvent evaporation, and the impact of nPSi at varying concentrations (0.1%, 0.5%, and 1% *m*/*v*) on the properties were studied, including morphological, physicochemical, and mechanical properties. Moreover, the drug release capabilities of composite films were evaluated using methylene blue (MB) as a model antibacterial drug and antibacterial assays were conducted against *S. aureus* and *E. coli* bacteria.

## 2. Materials and Methods

### 2.1. Materials

Polyvinyl alcohol (PVA) (Parteck SRP 80, Merck Chemical, Darmstadt, Germany), hydrochloric acid (HCl) (37%, J.T. Baker, Phillipsburg, NJ, USA), sodium hydroxide (NaOH) (ACS, Macron, Radnor Township, PA, USA), sodium bicarbonate (NaHCO_3_) (ACS, Fremont, OH, USA), glycerin (Alkofarma, Lima, Peru), starch (Scharlau, Barcelona, Spain), isopropyl alcohol (ACS, Fermont, OH, USA), hydrogen peroxide (H_2_O_2_) (30%, Emsure, Elk Grove Village, IL, USA) and ethanol (P.A., Emsure, Elk Grove Village, IL, USA) were obtained from Sigma-Aldrich, Saint Luis, MO, USA. 

### 2.2. Preparation of Bolaina Nanocellulose

To obtain the nanocellulose (NC), a suspension of 3% by weight of Bolaina was prepared and stirred for 24 h. Then, the pH was adjusted to 2 with a solution of HCl. The sample was washed with deionized water and resuspended to adjust the pH to between 4.5 and 5, followed by the addition of NaOH (5%) solution to neutralize the suspension and adjust the pH to 9.0. The suspension was bleached with a 3% (*v*/*v*) sodium hypochlorite solution. Finally, the suspension was washed with deionized water and adjusted to a conductivity of 2 μS/cm. It was then subjected to ball milling (YXQM-2L Planetary ball mill machine—Changsha Samy Instrument & Equipment Co., Ltd., Hunan, China).

### 2.3. Synthesis of Nanoporous Silicon Microparticles

Nanoporous silicon (nPSi) layers were fabricated by an electrochemical etching p+ type Si wafer in an HF (48%)/EtOH (1:2) solution. The applied current density was set to 80 mA/cm^2^ for 30 min. Afterward, an electropolishing pulse was applied to obtain free-standing nPSi layers. To facilitate the removal of the PSi layer from the Si substrate, a brief electropolishing pulse of 150 mA/cm^2^ for 2 s was administered. Next, diamond tips were used to scrape nPSi free-standing layers, resulting in the production of particles. Then, they were milled, collected in EtOH, and subjected to 20 min of ultrasonic agitation for homogenization. Suspended nPSi microparticles in the solution were collected by decanting followed by chemical oxidation using 30% (*v*/*v*) H_2_O_2_ for 12 h under orbital agitation. Then, the microparticles were rinsed with EtOH and dried.

### 2.4. Fabrication of Nanocellulose/Nanoporous Silicon Composite Films

The first step was the preparation of four solutions consisting of NaOH at a concentration of 50 g/L and starch at a quantity of 1 g. The mixture was stirred for 10 min at a temperature of 70 °C. Oxidized nPSi microparticles were added at various concentrations (0, 0.1, 0.5, and 1.0% *w*/*v*) and stirred at 70 °C for another 10 min. The agitation was then maintained for 12 h at room temperature.

The films were obtained in the second stage using the solvent evaporation method. To the previous solutions, 80 g of NC (2.4%) and 5 g of PVA (10%) were added and stirred for three hours at 80 °C. Then, 2 mL of isopropyl alcohol and 1.25 g of glycerol were added to each beaker. The mixtures obtained were stirred for 12 h to achieve homogeneity. Finally, the solutions were cast onto a glass Petri dish and allowed to dry for about 24 h at room temperature.

### 2.5. Physicochemical Characterizations

The optical properties analysis of samples were analyzed by UV–Vis absorbance spectra, which were recorded using a Jasco V-750 double-beam spectrophotometer (Tokyo, Japan). Photography of the samples was obtained on a Nikon D610 DSLR camera (Tokyo Japan). 

The morphology of the samples was observed by a variable pressure scanning electron microscope VPSEM, SU-3500, Hitachi (Tokyo, Japan), using an acceleration voltage of 10 kV. 

The chemical features of the samples were determined by Fourier transform infrared spectroscopy–attenuated total reflectance (FTIR–ATR, CARY 630 Technologies, Santa Clara, CA, USA) using direct transmittance. The samples were analyzed in the spectral region between 4000 cm^−1^ and 400 cm^−1^, with a resolution of 2 cm^−1^ and an average of 32 scans. 

The crystalline and semicrystalline structures of the samples were examined using the Rigaku X-ray diffractometer Smartlab model, with a goniometer Theta–Theta Bragg-Brentano geometry and the solid-state detector D/teX Ultra 250 model (Rigaku Corporation, Tokyo, Japan). The diffraction patterns were recorded using Cu-Ka (λ = 1.5418 Å) radiation at 40 kV/30 mA. The measurement was achieved between 5 and 60° (2θ), with a step of 0.02° and a scanning speed of 1°·min^−1^. Optical alignment was performed with NIST LaB6 (SRM 660c). The programs used for XRD data processing were Rigaku PDXL 2.7 with the ICDD PDF-5 2023 database for the qualitative part and TOPAS-Academic v6 for the refinement of the diffractograms by the Rietveld method. These programs were used to determine the percentage of crystallinity for each sample, the crystalline domain size, and the composition of the phases present. 

The thermal profiles of samples were measured using thermal gravimetric analysis (STA 6000, Perkin Elmer, Waltham, MA, USA). During the measurement, a weighted sample of about 6 mg was heated from 30 °C to 550 °C at a heating rate of 20 °C/min under a dry nitrogen atmosphere

### 2.6. Mechanical Analysis

Samples with dimensions of 20 mm long and 5 mm wide were used in sextuplicate on a Deben MICROTEST tensile stage with a 200 N load cell. During the execution of the test, the speed of the equipment was adjusted to 1.5 mm/min to obtain the modulus of elasticity (E, MPa), tensile strength (σ, MPa), and elongation at failure (ε, %) [15].

### 2.7. Drug Release Profiles

Samples were loaded with methylene blue (MB, Mw = 373.90 g/mol) using a 0.001 M solution (pH = 7) for 15 min at 50 RPM and room temperature, followed by three consecutive Milli-Q water rinse steps of 5, 3, and 1 min, respectively. 

To determine the release profiles, MB-loaded samples were placed into vials filled with PBS (pH = 7.4, 37 °C) in a horizontal shaker at 100 RPM (NB-2005 LN, Biotek, Winooski, VT, USA). At predetermined time intervals, MB was detected using a Thermo Scientific Evolution-220 spectrophotometer (Waltham, MA, USA) at 548 and 671 nm [10]. All experiments were conducted in triplicate. In order to determine the mechanism of drug release, the model monolithic solution model for slab geometries was fitted to the release profiles [16].

For short times, $\frac{M_{t}}{M_{\infty }}\leq 0.6\;as\;follows$:

(1) $$\frac{M_{t}}{M_{\infty }}=4{\left(\frac{Dt}{\pi L^{2}}\right)}^{1/2}$$

For late times, $\frac{M_{t}}{M_{\infty }}\geq 0.4\;as\;follows$:

(2) $$\frac{M_{t}}{M_{\infty }}=1-\frac{8}{\pi^{2}}exp\left(-\frac{\pi^{2}Dt}{L^{2}}\right)$$

where $M_{t}$ and $M_{\infty }$ denote the cumulative amounts of drug released at time *t* and at infinite time, respectively; *D* is the diffusion coefficient of the drug within the system; and *L* represents the total thickness of the film. Equation (1) can be used for just up to 60% of the released drug, and Equation (2) from 40% to 100% of the released drug. The fitting of the models was conducted with OriginPro 2022b software.

### 2.8. Antibacterial Assays

Firstly, samples without MB (control) and loaded with MB were sterilized with oxygen plasma in a Harrick Plasma Cleaner model PDC-32G (Ithaca, NY, USA). The equipment used a radio frequency power of 18 W and was operated under a 100 mL/min flow of O_2_ at a pressure of less than 0.2 mmHg for 7 min [17].

After sterilization, samples were cultured to assess the antimicrobial activity, employing the reference bacterial strains specified in the ISO 22196 standard [18]: *Staphylococcus aureus* (Gram-positive) and *Escherichia coli* (Gram-negative). The assessment was conducted using the modified Kirby–Bauer method [19,20,21]. Bacterial suspensions were prepared from pure cultures by harvesting the colonies using a sterile Drigalsky loop, followed by suspension in 50 mL of sterile-distilled water. The suspension was then homogenized for 10 min using a Vortex shaker. Subsequently, the preparation was diluted to achieve a bacterial concentration of 1.5 × 10^6^ CFU/mL, corresponding to 0.5 on the MacFarland turbidity scale. Each culture plate was inoculated with 200 µL of the bacterial suspension, which was allowed to air-dry for 20 min. Subsequently, the samples were applied to the surface of the plates. The plates were then incubated for 24–48 h at 35 ± 1 °C. Following the incubation period, the presence of inhibition zones around each sample was verified by measuring and recording them for both the control and samples loaded with MB. 

The cultures for each bacterial strain were replicated in triplicate, and the data were analyzed using an ANOVA test to determine if significant differences existed between the studied groups. Upon establishing the presence of significant differences, multiple comparisons were conducted using a post hoc test to specifically identify between which groups these differences were significant.

## 3. Results and Discussion

**Figure 1:**
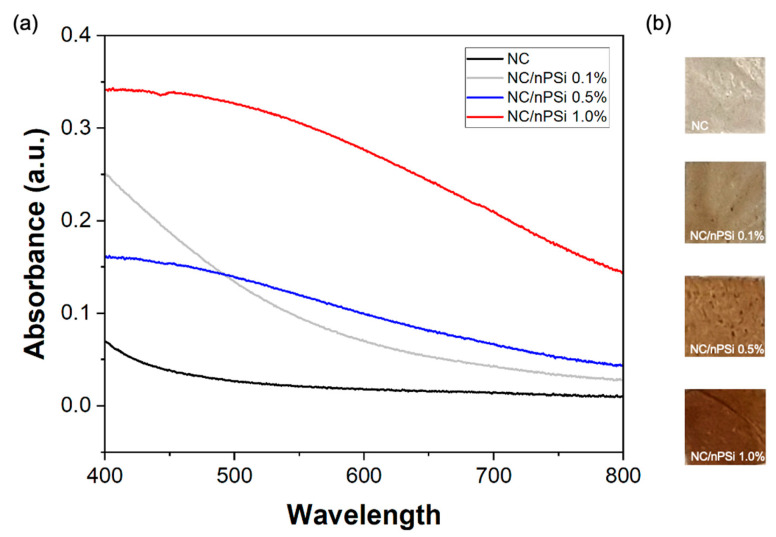
(**a**) UV–Vis spectra of samples and (**b**) representative photograph images of samples (0.5 × 0.5 cm^2^).

UV-Vis absorption spectra of NC and NC/nPSi films with different nPSi% were performed in the wavelength range from 400 to 800 nm. Figure 1a shows the absorbance spectra of the obtained films. The NC film showed minimal absorbance close to zero in the range of 450 to 800 nm, which was due to its high transparency in the visible range. The spectra of the NC/nPSi composite films showed an increase in absorbance as the concentration of nPSi in the films increased. This opacity of the films is attributed to the incorporation of nPSi microparticles [13]. The NC/nPSi 0.1% sample follows the same trend as the NC film but presents a significantly higher absorbance in the range of 400–500 nm. The NC/nPSi composite films with 0.5% and 1.0% nPSi exhibited similar trends in their spectra, but the sample with 1.0% nPSi showed higher absorbance (0.34) than the film with 0.5% nPSi (0.16) at 400 nm, reaching their minimum absorbance at 800 nm (0.14 and 0.044, respectively). Representative photographic images of the samples are exhibited in Figure 1b. They are a darker brown according to their increase in nPSi%, which is in concordance with UV-Vis analysis. 

**Figure 2:**
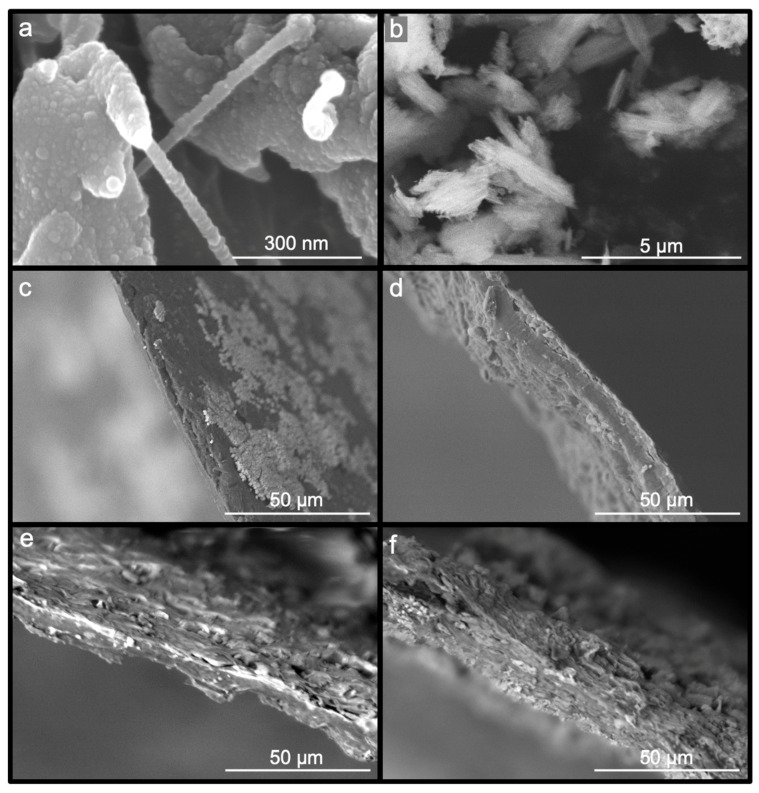
SEM images of samples: (**a**) NC recently milled, (**b**) nPSi microparticles, (**c**) NC film, (**d**) NC/nPSi 0.1%, (**e**) NC/nPSi 0.5%, and (**f**) NC/nPSi 1.0%.

The morphology of the samples was investigated using scanning electron microscopy (SEM). The nanofibers were synthesized from Bolaina wood through a process that resulted in gel suspension after passing through the ball mill. The presence of fibrils with diameters between 50 and 100 nm was confirmed through the SEM image shown in Figure 2a. Additionally, spherical particles were observed on the surface of smaller fibers. Figure 2b shows the nPSi microparticles, which had a spike-like structure with around a 0.5–1 μm width and a length ranging 1–3 μm. Moreover, microparticles are characterized by columnar-like pores with an average pore diameter of 50 nm. On the other hand, Figure 2c presents the NC as a film with a relatively homogeneous surface and a thickness of 6.5 ± 1 µm. The NC/nPSi films with nPSi concentrations of 0.1%, 0.5%, and 1% are shown in Figure 2d, Figure 2e, and Figure 2f, respectively. The films have thicknesses of 10.5 ± 2.5 µm, 12.7 ± 2.7 µm, and 29.5 ± 3.7 µm for samples at NC/nPSi 0.1%, NC/nPSi 0.5%, and NC/nPSi 1.0%, respectively. 

**Figure 3:**
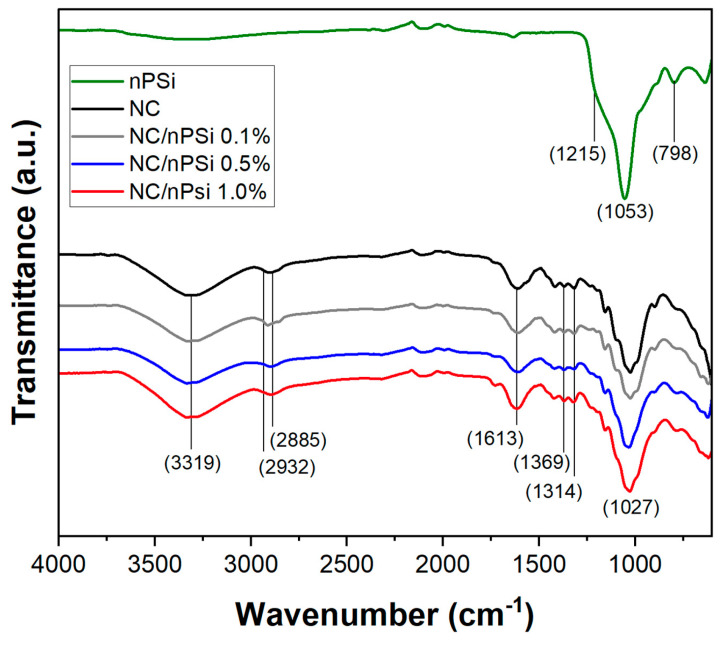
FT-IR spectra of nanocellulose, nPSi microparticles, and NC/nPSi composite films with different nPSi%.

FTIR-ATR analysis was performed to study the possible changes in the structural properties of NC, nPSi microparticles, and NC/nPSi composite films and their interactions among the components. The obtained spectra are shown in Figure 3. The nPSi spectrum showed a band at 798 cm^−1^, which is associated with the characteristic vibration of the Si-OH bond [22]. The band at 1053 cm^−1^ with a shoulder at 1215 cm^−1^ is attributed to the Si-O-Si stretching mode [23,24]. Both the NC and NC/nPSi samples exhibited a peak at 3319 cm^−1^, which is associated with the -OH stretching vibrations of cellulose. Additionally, this peak in the NC spectrum is related to the intramolecular hydrogen bonding of cellulose II [25,26]. A peak at 2885 cm^−1^ was also observed, which is attributed to the antisymmetric and symmetric vibration of the -CH_2_ groups. In addition, the appearance of a shoulder at approximately 2932 cm^−1^ was observed, which is associated with the C-H stretching vibrations of the alkane groups of the cellulose chain [27]. The peak at 1613 cm^−1^ is associated with -OH vending, which varies with the nanocellulose content [28]. Also, the peaks at 1369 and 1314 cm^−1^ are related to the vending modes CH and CH_2_ [29]. Finally, at 1027 cm^−1^, a peak attributable to the stretching of the C-O bond was observed, and this peak is also linked to the presence of non-hydrolyzed hemicellulose with the cellulose nanofibers [30].

**Figure 4:**
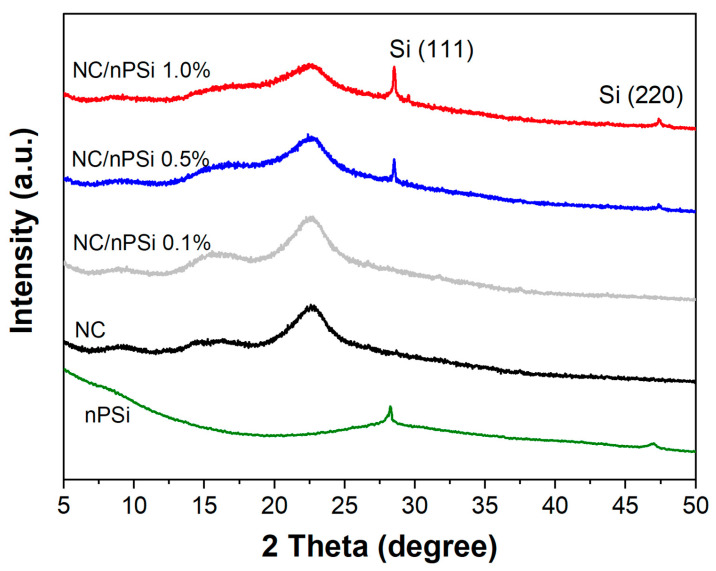
XRD analysis of NC/nPSi composite films with different nPSi%.

X-ray diffraction (XRD) was used to analyze the crystallinity of the nPSi, NC, and NC/nPSi films (Figure 4). The diffractogram of the NC revealed the presence of different peaks, including those located at 15.7° and 22.5°, corresponding to the 110 and 200 crystalline planes, respectively, which are characteristic of type Iβ-cellulose [31,32]. The diffractograms of nPSi microparticles exhibit characteristic peaks at 28.5° and 47.5°, respectively, which are associated with the (111) and (220) planes of Si (13). These peaks were also detected in the composite films containing 0.5% and 1% nPSi. The intensity of these peaks increased with the concentration of nPSi, and no high displacements were observed, indicating weak interactions (hydrogen bonds) between NC and nPSi.

On the other hand, X-ray diffraction profiles were optimized by TOPAS-Academic v6, using the pattern-fitting techniques by Rietveld refinement [33,34]. For the quantification of the semi-crystalline and amorphous phases of the nanocrystalline cellulose sample used as the matrix in the composite films, the crystallographic parameters obtained from the ICCD database were assumed for the profile refinements: monoclinic phase cellulose I beta (00-056-1718) and Triclinic Cellulose I Alpha (00-056-1719). On the other hand, for the quantification of the amorphous phase, the method of Pawley and LeBail was adapted in the adjustment, using a pseudo-one-dimensional orthorhombic phase in order to restrict the two main peaks of 2θ = 18.04° and 2θ = 20.64° [34]. The background was simulated using a Chebyshev polynomial function with 10 order levels. The peak shapes of the diffraction lines were defined by Gaussian and Lorentzian distributions, resulting in a goodness-of-fit of approximately 1.1. Additionally, monoclinic cellulose was corrected for preferential orientation in the 200 planes using the mathematical theory of March [35].

The results shown in Table 1 support the crystallographic structure of NC, indicating a predominantly monoclinic structure with crystalline domains of 10 nm. The alpha and beta phases have slightly smaller unit cells than those indicated in the database. Quantitative data are normal, and the patterns show an amorphous halo.

**Figure 5:**
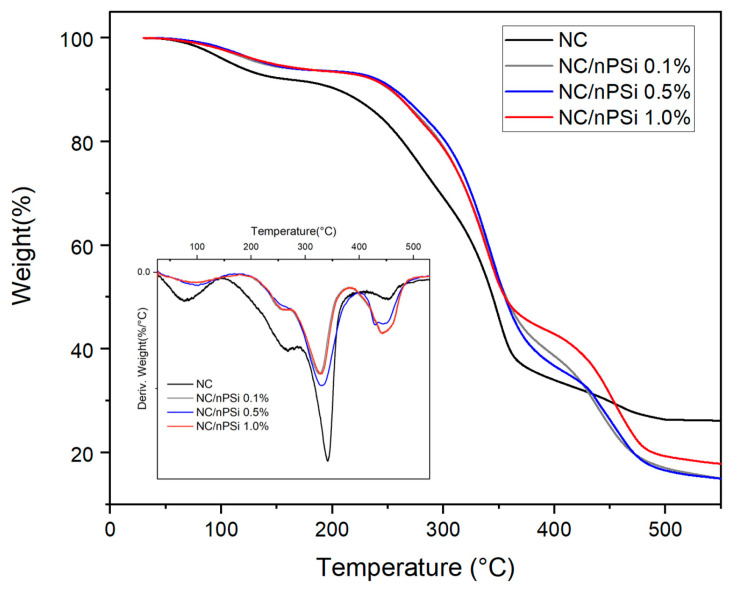
TGA and DTG curves (inset) of NC/nPSi films with different nPSi%.

Thermogravimetric analysis (TGA) was conducted to examine the thermal properties of NC and NC/nPSi films in terms of weight loss across a range of temperatures. Figure 5 illustrates that all films exhibit four distinct stages of degradation. The initial stage occurs between 50 and 100 °C, which corresponds to water evaporation, resulting from adsorbed moisture and water molecules trapped within the material [36]. The second stage occurs between 170 and 290 °C, which is associated with the degradation of the side chain associated with the C-C and C-O bonds of the polyvinyl alcohol (PVA) present in the films [37]. The third stage is associated with the decomposition of nanocellulose, with a Td of 341 °C, which consists of the cleavage of β-1-4 glycosidic bonds. Finally, the fourth stage, between 389 and 490 °C, corresponds to the general degradation of the PVA backbone composed only of carbon atoms [38].

The effect of nPSi on the thermal stability of the films was investigated. It was found that the NC/nPSi 0.1, 0.5, and 1.0% films exhibited higher thermal stability, as indicated by the T_5%_ (the temperature at which 5% of the mass is lost) values, which were 138, 143, and 146 °C, respectively, compared to that of NC, which was 110 °C. The T_50%_ (the temperature at which 50% of the mass was lost) of the nPSi films exhibited an increase of 10 °C with respect to the nanocellulose film (344 °C). Therefore, the incorporation of nPSi into nanocellulose films enhances the thermal properties of the material. Although the operating temperature range of the drug delivery system aligns with human body temperature (37 °C), the higher thermal stability of the obtained composite films (138–146 °C) ensures robustness, durability, and reliability, significantly enhancing the material’s overall performance and longevity. Furthermore, higher thermal stability correlates with improved durability and resistance to various forms of stress, including mechanical and chemical forms. This increased resilience can extend the material’s lifespan, even in applications involving lower temperatures. 

**Figure 6:**
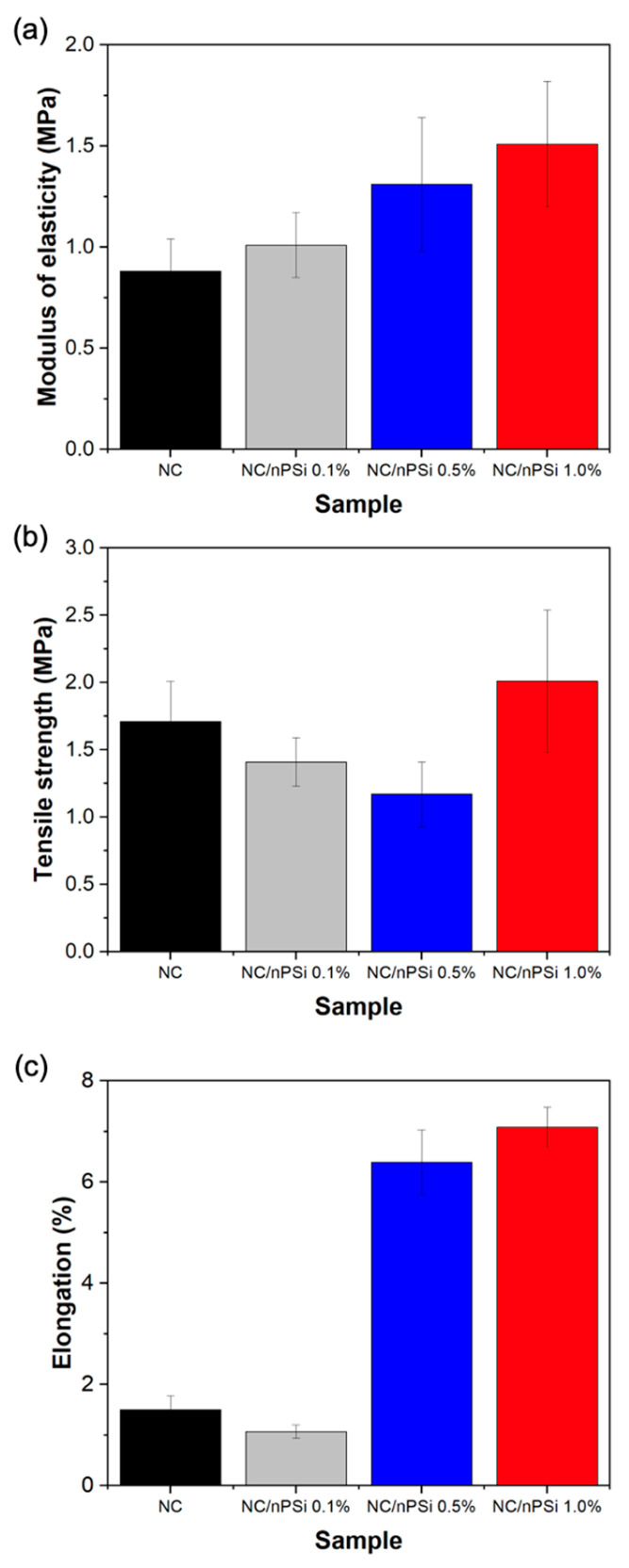
Mechanical analysis of NC and NC/nPSi composite films with different nPSi%: (**a**) modulus of elasticity, (**b**) tensile strength, and (**c**) elongation.

Uniaxial tensile tests were conducted to assess the mechanical properties of composite films fabricated with “bolaina” nanocellulose (NC) and nanoporous silicon (nPSi) microparticles. The results are illustrated in Figure 6a–c, depicting the elasticity modulus, tensile strength, and elongation, respectively. According to Figure 6a, the NC film exhibits an elastic modulus of 0.88 MPa. Notably, these values escalate from 1.01 to 1.51 MPa with increasing concentrations of nPSi in the films. It is noteworthy that the addition of 1% nPSi resulted in a remarkable 71.60% increase in the elastic modulus. Regarding tensile strength values, Figure 6b indicates that the presence of the nPSi-dispersed phase led to a reduction in this characteristic in the composite films. However, the sample containing the highest nPSi concentration (1%) demonstrated an increase in this property, rising from 1.41 to 2.01 MPa and representing a 17.54% increase compared to the pure NC film. In Figure 6c, a significant increase in elongation is evident with increasing proportions of nPSi in the composite films. While the NC sample exhibited only 1.5% elongation, films incorporating 0.1% to 1% of nPSi recorded elongation increases ranging from 1.07% to 7.08%, respectively. This represents a maximum 372% increase in percentage terms. Overall, the mechanical tests conducted on composite films demonstrated that the presence of nPSi positively influenced the mechanical properties of the samples. These findings suggest a direct correlation between the concentration of nPSi and the enhancement of these properties.

**Figure 7:**
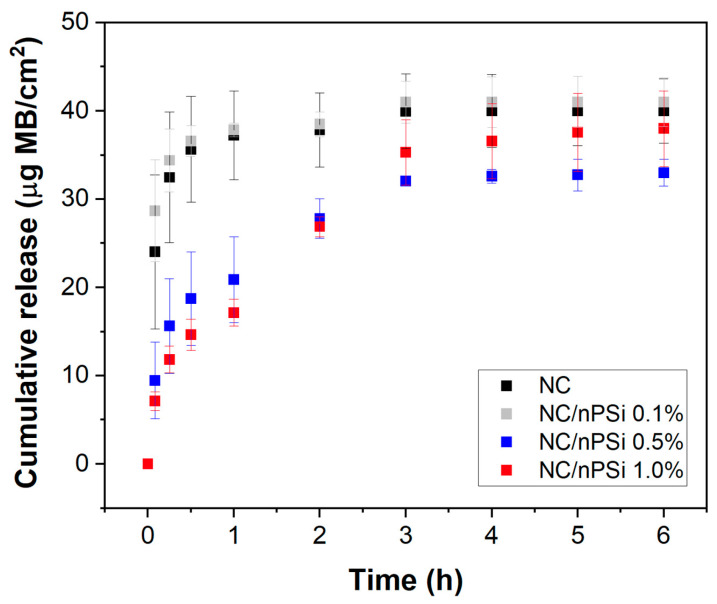
Methylene blue release profiles of NC/nPSi films with different nPSi%.

The capability of NC and NC/nPSi films to control drug release was evaluated using methylene blue dye (MB) as a model drug. According to Figure 7, the NC film and NC/nPSi 0.1% film released MB concentrations of 24 and 29 μg MB/cm^2^, respectively, after 1 h. In contrast, the NC/nPSi 0.5% and 1.0% films only released MB concentrations of 9.5 and 7.1 μg RB/cm^2^, respectively, after 1 h. These results suggest that concentrations higher than 0.1% nPSi can retain 30% more MB. The release profiles of NC and NC/nPSi at 0.1% were very similar, reaching a release equilibrium of 40 μg RB/cm^2^ after 3 h. After this time, there were no differences in the release profile of NC/nPSi 0.5% and 1.0% films, with 33 and 38 µg RB/cm^2^, respectively. The samples with nPSi could retain MB due to their physical deposition on nanoporous nPSi microparticles, which is associated with their high surface area and apparent solubility [39]. These properties enable a higher controlled release of drugs. Additionally, the hydroxyl groups of nanocellulose facilitate the retention of various drugs through weak interactions, such as hydrogen bonds. The formulation of this new material enhances the capabilities of NC and nPSi for the controlled delivery of drugs, such as MB, in a film format.

Considering the NC/nPSi composites as thin, homogeneous drug-containing films, the monolithic solution mathematical model can be utilized to determine the diffusion coefficients [40]. In this model, it is assumed that the initial drug concentration is lower than the drug solubility, and the drug is released solely via diffusion through the device. In this context, since the initial MB concentrations in composite films are considerably low, for the order of micrograms per milliliter, and the solubility of MB is 43.6 mg/mL [41], this condition is met. Table 2 presents the diffusion coefficient values obtained for slab geometries at short and late times using this model.

In the majority of cases, the results indicate that the monolithic solution model was well-fitted to the experimental release profiles for both short and late release times, thereby confirming diffusion as the primary mechanism of MB release. Additionally, the obtained diffusion coefficients were found to be inversely proportional to the % nPSi. This slower and more controlled release of MB in the samples could be attributed to the nanoporosity, high surface area, and large pore volume of nPSi within the composite films [42], which are particularly evident in the samples NC/nPSi 0.5% and NC/nPSi 1.0%.

**Figure 8:**
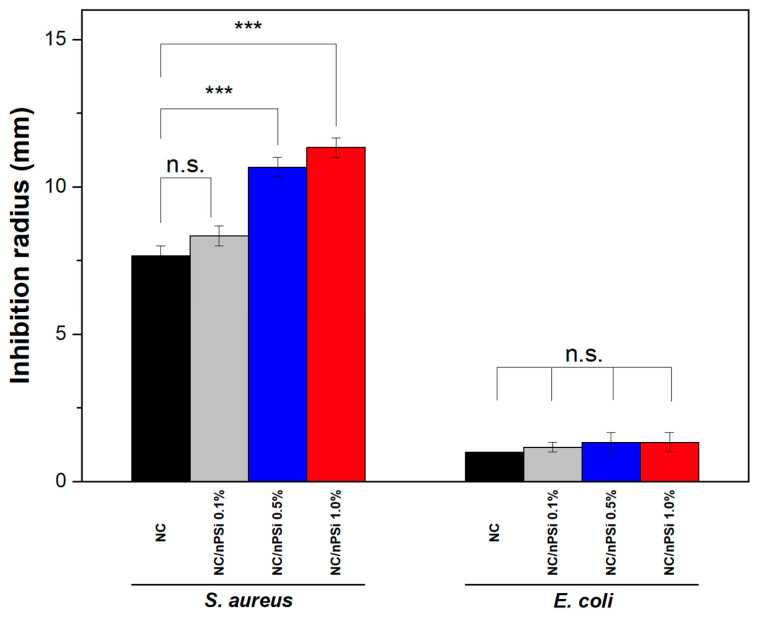
Inhibition radius of NC and NC/nPSi composite films loaded with methylene blue against *Staphylococcus aeureus* and *Escherichia coli*. Significant differences are presented as *** is *p*-value ≤ 0.001, and ns means not statistically significant.

The antibacterial activity of NC and NC/nPSi composite films loaded with and without MB was tested against *Staphylococcus aureus* (Gram-positive) and *Escherichia coli* (Gram-negative) using the diffusion method. The control samples, composite films without MB, did not present any antibacterial effect, and both types of bacteria spread over the entire surface of the culture medium. On the other hand, the results indicated that NC and NC/nPSi samples loaded with MB were effective in inhibiting the growth of bacteria (Figure 8). In this sense, the largest inhibition halos were observed against *S. aureus*: NC and NC/nPSi 0.1% showed an inhibition halo at around 8 mm, while the NC/nPSi films with 0.5% and 1.0% nPSi presented a higher inhibition halo of 11 mm. It is worth noting that the observed difference between the samples is significant. On the other hand, for the case of *E. coli* bacteria, all samples showed only around 1 mm of inhibition halo, and no significant difference was observed between them. These results support the findings of previous works in which Gram-positive bacteria are more vulnerable to nPSi than Gram-negative bacteria [43]. Additionally, the authors attribute the antibacterial properties of nPSi to their ability to denature microbial proteins and interfere with bacterial DNA replication.

## 4. Conclusions

The Bolaine nanocellulose film with nanoporous silica nanoparticles was obtained by film casting. The effect of different concentrations of nPSi (0.1%, 0.5%, and 1.0% *w*/*v*) on the properties of nanocellulose films was investigated by means of physicochemical, morphological, and mechanical analysis. The presence of nPSi in NC/nPSi films and weak interactions between nanocellulose hydroxyl groups and Si were confirmed by XRD. It was determined that higher concentrations of nPSi could lead to increased opacity, thermal stability, and mechanical properties. Specifically, NC/nPSi samples exhibited higher decomposition temperatures, elastic moduli, and elongation percentages compared to NC films. Additionally, both NC and NC/nPSi films loaded with MB demonstrated antibacterial activity, effectively inhibiting the growth of *E. coli* and *S. aureus*. In particular, the NC/nPSi 0.5% and 1.0% film showed a significant effect against *S. aureus*. It was also found that the release of MB followed a monolithic solution model, and the diffusion coefficients were inversely proportional to % nPSi. Therefore, its incorporation into the material allowed for a more controlled release. The results demonstrate the production of an NC/nPSi film as a controlled drug release system.

## Figures and Tables

**Table 1:** Rietveld refinement results for crystalline nanocellulose.

	Experimental			ICDD Data Card	
Cellulose I	Alpha	Beta	Amorphous	Alpha	Beta
				(00-056-1719)	(00-056-1718)
Crystal system	Triclinic	Monoclinic	-	Triclinic	Monoclinic
Proportions (%)	26	67	7		
Crystalline domain (nm)	3	10			
Crystal parameters (Å)					
a	6.4	7.66		6.74	7.784
b	5.77	7.88		5.93	8.201
c	11.13	10.56		10.36	10.38
α	118.95			117	
β	111.3			113	
γ	82.91	96.18		81	96.5

**Table 2:** Diffusion coefficient values of samples obtained by the monolithic solution for slab geometry at short times and late times. In vitro release kinetics of methylene blue were performed in PBS at 37 °C.

Sample	Monolithic Solution			
	$\mathrm{Short}\;\mathrm{Times}\;\frac{M_{t}}{M_{\infty }}\leq 0.6$		$\mathrm{Late}\;\mathrm{Times}\;\frac{M_{t}}{M_{\infty }}\geq 0.4$	
	D (µm^2^/h)	R_adj_	D (µm^2^/h)	R_adj_
NC	29.3564	0.9428	28.5525	0.8593
NC/nPSi 0.1%	14.0012	0.9428	17.5981	0.6116
NC/nPSi 0.5%	17.2703	0.8857	16.47317	0.9344
NC/nPSi 1.0%	10.2231	0.9688	11.8551	0.9204

## Data Availability

Data are contained within the article.

## References

[1] Kargarzadeh H., Mariano M., Gopakumar D., Ahmad I., Thomas S., Dufresne A., Huang J., Lin N. (2018). Advances in cellulose nanomaterials. Cellulose.

[2] Mandal A., Chakrabarty D. (2014). Studies on the mechanical, thermal, morphological and barrier properties of nanocomposites based on poly(vinyl alcohol) and nanocellulose from sugarcane bagasse. J. Ind. Eng. Chem..

[3] Pennells J., Godwin I.D., Amiralian N., Martin D.J. (2020). Trends in the production of cellulose nanofibers from non-wood sources. Cellulose.

[4] Djafari Petroudy S.R., Shojaeiarani J., Chabot B. (2023). Recent advances in isolation, characterization, and potential applications of nanocellulose-based composites: A comprehensive review. J. Nat. Fibers.

[5] Kupnik K., Primožič M., Kokol V., Leitgeb M. (2020). Nanocellulose in drug delivery and antimicrobially active materials. Polymers.

[6] Huo Y., Liu Y., Xia M., Du H., Lin Z., Li B., Liu H. (2022). Nanocellulose-based composite materials used in drug delivery systems. Polymers.

[7] Rivera-Hernández G., Antunes-Ricardo M., Martínez-Morales P., Sanchez M.L. (2021). Polyvinyl alcohol based-drug delivery systems for cancer treatment. Int. J. Pharm..

[8] Varghese R.T., Cherian R.M., Chirayil C.J., Antony T., Kargarzadeh H., Thomas S. (2023). Nanocellulose as an avenue for drug delivery applications: A mini-review. J. Compos. Sci..

[9] Colturato P.L., Goveia D. (2022). Controlled release of vitamin D3 using a nanocellulose-based membrane. Sci. Rep..

[10] Hernández-Montelongo J., Oria L., Cardenas A.B., Benito N., Romero-Sáez M., Recio-Sánchez G. (2018). Nanoporous silicon composite as potential system for sustained delivery of florfenicol drug. Phys. Status Solidi Basic Res..

[11] Li W., Liu Z., Fontana F., Ding Y., Liu D., Hirvonen J.T., Santos H.A. (2018). Tailoring porous silicon for biomedical applications: From drug delivery to cancer immunotherapy. Adv. Mater..

[12] Capek I. (2020). Smart therapeutic-polymer-loaded silica nanomaterials. Nanomedicine. Polym.-Plast. Technol. Mater..

[13] França C.G., Plaza T., Naveas N., Santana M.H.A., Manso-Silván M., Recio G., Hernandez-Montelongo J. (2021). Nanoporous silicon microparticles embedded into oxidized hyaluronic acid/adipic acid dihydrazide hydrogel for enhanced controlled drug delivery. Microporous Mesoporous Mater..

[14] Cornelius J.P., Pinedo-Ramírez R., Sotelo Montes C., Ugarte-Guerra L.J., Weber J.C. (2018). Efficiency of early selection in Calycophyllum spruceanum and Guazuma crinita, two fast-growing timber species of the Peruvian Amazon. Can. J. For..

[15] Vergara-Figueroa J., Erazo O., Pesenti H., Valenzuela P., Fernández-Pérez A., Gacitúa W. (2023). Development of Thin Films from Thermomechanical Pulp Nanofibers of Radiata Pine (*Pinus radiata* D. Don) for Applications in Bio-Based Nanocomposites. Fibers.

[16] Siepmann J., Siepmann F. (2012). Modeling of diffusion controlled drug delivery. J. Control. Release.

[17] Moreira A.J., Mansano R.D., Pinto T.d.J.A., Ruas R., da Silva Zambon L., da Silva M.V., Verdonck P.B. (2004). Sterilization by oxygen plasma. Appl. Surf. Sci..

[18] (2011). Measurement of Antibacterial Activity on Plastics and Other Non-Porous Surfaces.

[19] Pujiastuti D., Palupi C. (2018). Perbandingan efektivitas antibakteri minyak atsiri bawang putih (Allium Sativum) dan black garlic terhadap bakteri Staphylococcus aureus dan Escherichia coli dengan metode Kirby-Bauer. J. Pharm. Sci. Med. Res..

[20] Trisia A., Philyria R., Toemon A.N. (2018). Uji Aktivitas Antibakteri Ekstrak Etanol Daun Kalanduyung (*Guazuma ulmifolia* Lam.) terhadap Pertumbuhan *Staphylococcus aureus* dengan Metode Difusi Cakram (Kirby-Bauer): Antibacterial Activity Test of Ethanol Extract from Kalanduyung Leaf (*Guazuma ulmifolia* Lam.) on Staphylococcus aureus Growth with Difussion Method (Kirby-Bauer). Anterior J..

[21] Schiller H., Young C., Schulze S., Tripepi M., Pohlschroder M. (2022). A Twist to the kirby-bauer disk diffusion susceptibility test: An accessible laboratory experiment comparing haloferax volcanii and escherichia coli antibiotic susceptibility to highlight the unique cell biology of archaea. J. Microbiol. Biol. Educ..

[22] Naveas N., Costa V.T., Gallach D., Hernandez-Montelongo J., Palma R.J.M., Garcia-Ruiz J.P., Manso-Silván M. (2012). Chemical stabilization of porous silicon for enhanced biofunctionalization with immunoglobulin. Sci. Technol. Adv. Mater..

[23] Ogata Y.H. (2018). Characterization of porous silicon by infrared spectroscopy. Handbook of Porous Silicon.

[24] Ellerbrock R., Stein M., Schaller J. (2022). Comparing amorphous silica, short-range-ordered silicates and silicic acid species by FTIR. Sci. Rep..

[25] Morán J.I., Alvarez V.A., Cyras V.P., Vázquez A. (2008). Extraction of cellulose and preparation of nanocellulose from sisal fibers. Cellulose.

[26] Zuluaga R., Putaux J.L., Cruz J., Vélez J., Mondragon I., Gañán P. (2009). Cellulose microfibrils from banana rachis: Effect of alkaline treatments on structural and morphological features. Carbohydr. Polym..

[27] Roy S., Rhim J.W. (2020). Carboxymethyl cellulose-based antioxidant and antimicrobial active packaging film incorporated with curcumin and zinc oxide. Int. J. Biol. Macromol..

[28] Hasanin M.S., Youssef A.M. (2022). Ecofriendly bioactive film doped CuO nanoparticles based biopolymers and reinforced by enzymatically modified nanocellulose fibers for active packaging applications. Food Packag. Shelf Life.

[29] Heidari H. (2018). Ag nanoparticle/nanofibrillated cellulose composite as an effective and green catalyst for reduction of 4-nitrophenol. J. Clust. Sci..

[30] Tibolla H., Pelissari F.M., Menegalli F.C. (2014). Cellulose nanofibers produced from banana peel by chemical and enzymatic treatment. LWT-Food Sci. Technol..

[31] Thulluri C., Balasubramaniam R., Velankar H.R. (2021). Generation of highly amenable cellulose-Iβ via selective delignification of rice straw using a reusable cyclic ether-assisted deep eutectic solvent system. Sci. Rep..

[32] Willhammar T., Daicho K., Johnstone D.N., Kobayashi K., Liu Y., Midgley P.A., Bergstrom L., Saito T. (2021). Local crystallinity in twisted cellulose nanofibers. ACS Nano.

[33] Young R. (1993). The Rietveld Method.

[34] Dinnebier R.E., Leineweber A., Evans J.S.O. (2018). Rietveld Refinement: Practical Powder Diffraction Pattern Analysis Using TOPAS.

[35] March A. (1932). Mathematische Theorie der Regelung nach der Korngestah bei affiner Deformation. Z. Krist. Cryst. Mater..

[36] Garrido-Miranda K.A., Rivas B.L., Pérez -Rivera M.A., Sanfuentes E.A., Peña-Farfal C. (2018). Antioxidant and antifungal effects of eugenol incorporated in bionanocomposites of poly(3-hydroxybutyrate)-thermoplastic starch. LWT-Food Sci. Technol..

[37] Suganthi S., Vignesh S., Kalyana Sundar J., Raj V. (2020). Fabrication of PVA polymer films with improved antibacterial activity by fine-tuning via organic acids for food packaging applications. Appl. Water Sci..

[38] Panda P.K., Dash P., Biswal A.K., Chang Y.H., Misra P.K., Yang J.M. (2022). Synthesis and characterization of modified poly(vinyl alcohol) membrane and study of its enhanced water-induced shape-memory behavior. J. Polym. Environ..

[39] Riikonen J., Xu W., Lehto V.P. (2015). Porous silicon in drug delivery applications. Electrochemically Engineered Nanoporous Materials.

[40] Siepmann J., Lecomte F., Bodmeier R. (1999). Diffusion-controlled drug delivery systems: Calculation of the required composition to achieve desired release profiles. J. Control. Release.

[41] Khan I., Saeed K., Zekker I., Zhang B., Hendi A.H., Ahmad A., Ahmad S., Zada N., Ahmad H., Shah L.A. (2022). Review on methylene blue: Its properties, uses, toxicity and photodegradation. Water.

[42] Rocha-García D., Guerra-Contreras A., Rosales-Mendoza S., Palestino G. (2016). Role of porous silicon/hydrogel composites ondrug delivery. Open Mater. Sci..

[43] Jabir M.S., Nayef U.M., Jawad K.H., Taqi Z.J., Buthenhia H., Ahmed N.R. (2018). Porous silicon nanoparticles prepared via an improved method: A developing strategy for a successful antimicrobial agent against Escherichia coli and Staphylococcus aureus. IOP Conf. Ser. Mater. Sci. Eng..

